# Paradoxical response to chest wall loading predicts a favorable mechanical response to reduction in tidal volume or PEEP

**DOI:** 10.1186/s13054-022-04073-2

**Published:** 2022-07-05

**Authors:** John Selickman, Pierre Tawfik, Philip S. Crooke, David J. Dries, Jonathan Shelver, Luciano Gattinoni, John J. Marini

**Affiliations:** 1 Department of Pulmonary and Critical Care Medicine, University of Minnesota School of Medicine, 640 Jackson Street, Mail Stop 11203B, MinneapolisSt. Paul, MN 55101 USA; 2 Department of Mathematics, Vanderbilt University, Nashville, TN USA; 3 Department of Surgery, Regions Hospital, St Paul, MN USA; 4 Department of Surgery, University of Minnesota School of Medicine, Minneapolis, MN USA; 5 Department of Pulmonary and Critical Care Medicine, Methodist Hospital, St. Louis Park, MN USA; 6 Department of Anesthesiology, Medical University of Göttingen, University Medical Center Göttingen, Göttingen, Germany; 7 Department of Critical Care Medicine, Regions Hospital, St. Paul, MN USA

## Abstract

**Background:**

Chest wall loading has been shown to paradoxically improve respiratory system compliance (C_RS_) in patients with moderate to severe acute respiratory distress syndrome (ARDS). The most likely, albeit unconfirmed, mechanism is relief of end-tidal overdistension in ‘baby lungs’ of low-capacity. The purpose of this study was to define how small changes of tidal volume (V_T_) and positive end-expiratory pressure (PEEP) affect C_RS_ (and its associated airway pressures) in patients with ARDS who demonstrate a paradoxical response to chest wall loading. We hypothesized that small reductions of V_T_ or PEEP would alleviate overdistension and favorably affect C_RS_ and conversely, that small increases of V_T_ or PEEP would worsen C_RS_.

**Methods:**

Prospective, multi-center physiologic study of seventeen patients with moderate to severe ARDS who demonstrated paradoxical responses to chest wall loading. All patients received mechanical ventilation in volume control mode and were passively ventilated. Airway pressures were measured before and after decreasing/increasing V_T_ by 1 ml/kg predicted body weight and decreasing/increasing PEEP by 2.5 cmH_2_O.

**Results:**

Decreasing either V_T_ or PEEP improved C_RS_ in all patients. Driving pressure (DP) decreased by a mean of 4.9 cmH_2_O (supine) and by 4.3 cmH_2_O (prone) after decreasing V_T_, and by a mean of 2.9 cmH_2_O (supine) and 2.2 cmH_2_O (prone) after decreasing PEEP. C_RS_ increased by a mean of 3.1 ml/cmH_2_O (supine) and by 2.5 ml/cmH_2_O (prone) after decreasing V_T._ C_RS_ increased by a mean of 5.2 ml/cmH_2_O (supine) and 3.6 ml/cmH_2_O (prone) after decreasing PEEP (*P* < 0.01 for all). Small increments of either V_T_ or PEEP worsened C_RS_ in the majority of patients.

**Conclusion:**

Patients with a paradoxical response to chest wall loading demonstrate uniform improvement in both DP and C_RS_ following a reduction in either V_T_ or PEEP, findings in keeping with prior evidence suggesting its presence is a sign of end-tidal overdistension. The presence of ‘paradox’ should prompt re-evaluation of modifiable determinants of end-tidal overdistension, including V_T_, PEEP, and body position.

**Supplementary Information:**

The online version contains supplementary material available at 10.1186/s13054-022-04073-2.

## Background

Airway driving pressure (DP) is used routinely to guide ‘lung-protective’ ventilation in acute respiratory distress syndrome (ARDS). The effect of tidal volume (V_T_) on DP is determined by tidal compliance of the integrated respiratory system (C_RS_), which is comprised of the lungs and chest wall. For a fixed V_T_ and positive end-expiratory pressure (PEEP), any net change in C_RS_ alters DP in the opposite direction. Because they share a common volume, a decrease in compliance of either the lungs (C_L_) or the chest wall (C_CW_) simultaneously decreases C_RS_ unless there is a compensatory improvement in its counterpart.

In recent work, reducing lung volume and C_CW_ by external compression, or ‘loading’, has been noted to improve C_RS_ in patients with moderate to severe ARDS [1–6]; by immediate implication, loading must therefore result in improved C_L_ and lower transpulmonary pressure in these patients. The most appealing (but as yet unconfirmed) explanation for this counterintuitive mechanical ‘paradox’ (i.e., decreased C_CW_ resulting in improved C_RS_) is relief of end-tidal overdistension that occurs in the unloaded state.

The purpose of this study was to define how choice of V_T_ and PEEP affect C_RS_ in ARDS patients demonstrating a paradoxical response to chest wall loading. When V_T_ operates in the linear (‘middle’) portion of the pressure–volume relationship, as intended for lung protection, small changes of V_T_ and/or PEEP should leave C_RS_ unaffected [7]. On the other hand, assuming the underlying mechanism of the loading paradox is relief of end-tidal overdistension, we reasoned that ‘paradox positive’ patients would demonstrate a disproportionate reduction in DP (and by extension, increased C_RS_) following a small decrease of V_T_ or PEEP, both of which alleviate end-tidal overdistension. Conversely, we reasoned that such patients would demonstrate a disproportionate increase in DP (and decreased C_RS_) following a small increase of V_T_ or PEEP—changes which would exacerbate any end-tidal overdistension.

## Methods

This prospective, multi-center physiologic study was performed in two medical intensive care units (Regions Hospital, St. Paul MN, USA and Methodist Hospital, Minneapolis, MN, USA), with all data collected by the same investigative team between December 2021 and March 2022.

### Patients

Consecutive patients with ARDS (as defined by the Berlin consensus criteria [8]) who demonstrated no signs of active breathing were enrolled and evaluated. All received invasive mechanical ventilation under controlled conditions, with passive breathing assured by either ongoing administration of neuromuscular blockers or deep sedation sufficient to suppress any evidence of active breathing.

### Ventilatory strategy

All patients received mechanical ventilation in volume regulated, control mode (decelerating flow profile) using one of two ventilators: Puritan Bennett 980 (Medtronic; Carlsbad, California, USA) or Maquet Servo-I (Siemens; Bloomfield, Connecticut, USA). Baseline measurements were performed using the V_T_, PEEP and respiratory rate already prescribed by the clinical team for routine management prior to study enrollment.

### Measurements

Measurements were performed in the position of care, either supine or prone, and this was not altered for the purposes of data collection. When possible, study measurements were repeated in the opposite position within 24 h, provided that the criteria for passive breathing were still met.

In the supine orientation, measurements were performed in a semi-recumbent position with the head elevated to 30°; in the prone orientation, measurements were performed with the bed flat (0°). The highest airway pressure during inflation was recorded as the peak pressure. Plateau pressure was measured at least two seconds after performing an end-inspiratory pause. Total PEEP (the sum of set PEEP and auto-PEEP) was measured at least three seconds after performing an end-expiratory pause, assuring that zero flow was achieved.

Measurement of tidal airway pressures were repeated after the following interventions: (1) increasing V_T_ by 1 mL/kg predicted body weight (PBW); (2) decreasing V_T_ by 1 mL/kg PBW; (3) increasing PEEP by 2.5 cmH_2_O; and (4) decreasing PEEP by 2.5 cmH_2_O.

### Procedure for chest wall loading to detect paradoxical mechanical response

Following baseline measurements (obtained in an unloaded state), manual loading of the chest wall was performed. In the supine position, loading was accomplished by placing a hand over the patient’s umbilicus perpendicular to the axis between the xiphoid process and the pubis; in the prone position, the hand was placed at the approximate mid-point between the inferior costal margin and the iliac crest, perpendicular to the lumbar spine. To gauge load adequacy, an end-inspiratory hold was then performed and manual pressure applied until there was an upward deflection of the pressure–time waveform of ≥ 2 cmH_2_O, at which point chest wall loading was considered sufficient to influence transpulmonary pressure during tidal breathing. The inspiratory hold on the ventilator was then released, while continuing to apply sustained manual pressure on the abdomen or lumbar region. After five breaths had been delivered, measurements of tidal airway pressure were repeated, and manual pressure was then released.

#### Statistical analysis

A normality test was performed for all samples to verify a normal distribution. When normality was confirmed, the paired t-test was used to compare mean values of DP and C_RS_ at baseline, during chest wall loading, and following alteration of ventilator parameters as outlined above. In all instances where the normality assumption was not satisfied results were confirmed using the Mann–Whitney test. Differences at the level of a two-tailed *P* value < 0.05 were considered statistically significant.

## Results

Nineteen patients with ARDS were studied, of whom seventeen had ARDS secondary to novel coronavirus (C-ARDS). Seventeen demonstrated a paradoxical response to chest wall loading. Of these, paired measurements were obtained in both the supine and prone positions in eight; in the remaining nine patients, five were evaluated in only the supine position, and four were evaluated in only the prone position. All but one patient had either moderate or severe ARDS, all were ventilated in accordance with lung protective principles for ventilation, and none received extracorporeal support for gas exchange or hemodynamics (Table 1). Mortality at thirty days from the time of data acquisition was 70.6% (12/17). Ventilator settings and gas exchange at baseline are reported in Table 2.

**Table 1:** Patient characteristics

Subject	Diagnosis	Age (years)	Gender	BMI (kg/m^2^)	LOH (days)	LOI (days)	NMB	30-day survival
1	C-ARDS	59	Male	35	9	1	Y	N
2	C-ARDS	45	Female	31.5	22	14	N	Y
3	C-ARDS	55	Male	25.2	24	5	Y	N
4	C-ARDS	65	Male	25.2	26	23	N	N
5	ARDS	50	Female	23.5	15	13	Y	Y
6	C-ARDS	54	Male	28.5	14	14	N	Y
7	C-ARDS	70	Male	37.4	11	11	N	N
8	C-ARDS	47	Male	28.9	50	41	Y	N
9	ARDS	67	Female	25.3	1	2	Y	N
10	C-ARDS	70	Female	42.7	1	1	Y	N
11	C-ARDS	76	Male	31.7	11	2	N	N
12	C-ARDS	54	Male	29.2	10	1	Y	N
13	C-ARDS	62	Male	26.9	14	4	N	N
14	C-ARDS	30	Male	29.6	22	14	Y	Y
15	C-ARDS	63	Male	29.4	1	1	N	Y
16	C-ARDS	76	Female	30.1	13	6	N	N
17	C-ARDS	61	Male	28.8	41	37	Y	N
Mean		59.1		29.9	16.8	11.2		
SD		11.9		4.8	13.3	12.3		

*BMI* Body mass index, *LOH* Duration of hospitalization, *LOI* Duration of intubation, *NMB* Neuromuscular blockade, *C-ARDS* COVID-related acute respiratory distress syndrome, *ARDS* Acute respiratory distress syndrome, *Y* Yes, *N* No, SD Standard deviation

**Table 2:** Ventilator settings and gas exchange at baseline

Subject	Pa_O2_/Fi_O2_	Pa_CO2_ (mmHg)	V_T_ (mL/kg PBW)	PEEP set (cmH_2_O)	RR	Ventilatory ratio
1	85	50.8	7	16	22	2.1
2	137.5	101	4.9	12	30	4.0
3	109.3	66.7	6	7.5	24	2.6
4	83	81.3	5.4	6	32	3.7
5	87	62.6	3.9	6	27	1.8
6	157.2	65.3	5.9	9	32	3.3
7	245	63.4	5.5	12	22	2.0
8	82.2	63.2	5.9	5	30	3.0
9	86.9	61.5	5.4	8	32	2.8
10	147	48.1	7	14	34	3.1
11	191.4	47.4	5.9	12	28	2.1
12	160	74.3	6.6	10	24	3.1
13	98.3	49	4.6	10	32	1.9
14	102.5	45	5.2	8	28	1.7
15	65.6	59	4.6	12	34	2.5
16	185.5	58.7	6.7	12	26	2.7
17	73.4	92.7	4.7	6	34	4.0
Mean	123.3	64.1	5.6	9.7	28.9	2.7
SD	50.7	15.7	0.9	3.2	4.2	0.7

*Pa*_*O2*_ Partial pressure of arterial oxygen, *Fi*_*O2*_ Fraction of inspired oxygen, *Pa*_*CO2*_ Partial pressure of arterial carbon dioxide, *V*_*T*_ Tidal volume, *PEEP* Positive end-expiratory pressure, *RR* Respiratory rate, *SD* Standard deviation. Ventilatory ratio is defined as [minute ventilation (mL/min) x Pa_CO2_ (mmHg)]/(predicted body weight × 100 × 37.5)

### Chest wall loading

**Fig. 1:**
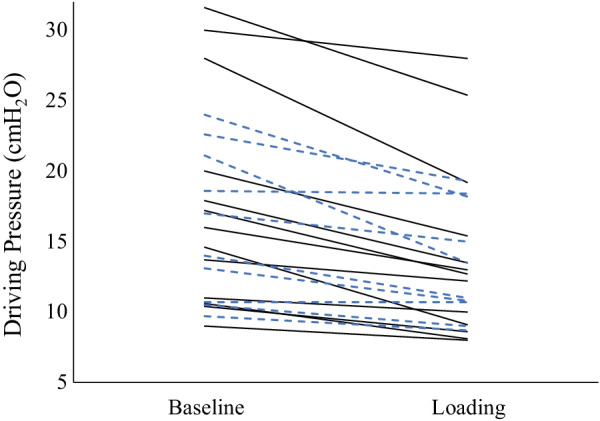
Individual changes in driving pressure at baseline and following chest wall loading. Patients in both the supine (black) and prone (blue dash) positions are represented

Chest wall loading reduced DP by a mean of 3.6 ± 2.3 cmH_2_O in the supine position and by 2.7 ± 2.3 cmH_2_O in the prone position (*P* < 0.01 for both). C_RS_ improved following chest wall loading by a mean of 6.1 ± 3.5 mL/cmH_2_O in the supine position and 4.1 ± 3.0 mL/cmH_2_O in the prone position (P ≤ 0.001 for both) (Table 3 and Fig. 1).

**Table 3:** Response to chest wall loading, decreased tidal volume, and decreased positive end-expiratory pressure Baseline measurements were performed (column A) followed by chest wall loading (column B). In the unloaded state, V_T_ was decreased by 1 mL/kg PBW (column C) and PEEP was decreased by 2.5 cmH2O (column D) in all seventeen patients.

		Baseline (A)	Loading (B)	↓ V_T_ (C)	↓ PEEP (D)	A to B (p value)	A to C (p value)	A to D (p value)
Supine	DP (cmH_2_O)	17.7 ± 7.7	14.1 ± 6.5	12.8 ± 5.4	14.8 ± 6.5	0.0001	< 0.0001	0.0001
	C_RS_ (mL/cmH_2_O)	25.7 ± 11.8	31.8 ± 13.2	28.9 ± 13	30.9 ± 14.6	< 0.0001	0.0002	0.0002
Prone	DP (cmH_2_O)	17.2 ± 6.2	14.5 ± 5.2	11.9 ± 3.5	15.0 ± 5.5	0.003	0.0001	0.008
	C_RS_ (mL/cmH2O)	24 ± 10.8	28.1 ± 11.8	28.1 ± 10.8	27.6 ± 11	0.001	0.005	0.004

*DP* Driving pressure, *C*_*RS*_ Respiratory system compliance, *V*_*T*_ Tidal volume, *PEEP* Positive end-expiratory pressure

### Decreasing V_T_ and PEEP in the unloaded state.

**Fig. 2:**
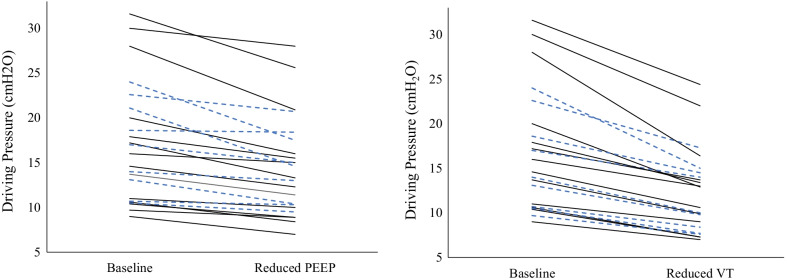
Changes in driving pressure by intervention. Individual changes in driving pressure following reduction in PEEP by 2.5 cmH_2_O (left panel) and tidal volume by 1 ml/kg PBW (right panel)*.* Patients in both the supine (black) and prone (blue dash) positions are represented. Graph is truncated at 5 cmH_2_O for viewing purposes

Decreasing V_T_ resulted in a reduced DP and an improved C_RS_ in all seventeen patients. DP decreased by a mean of 4.9 ± 2.8 cmH_2_O in the supine position and 4.3 ± 2.2 cmH_2_O in the prone position (*P* ≤ 0.001 for both). C_RS_ improved by a mean of 3.1 ± 2.1 mL/cmH_2_O in the supine position and 2.5 ± 2.1 mL/cmH_2_O in the prone position (*P* ≤ 0.005 for both) (Table 3 and Fig. 2).

Decreasing PEEP similarly resulted in reduced DP and improved C_RS_ in all seventeen patients. DP decreased by a mean of 2.9 ± 1.9 cmH_2_O in the supine position and 2.2 ± 2.2 cmH_2_O in the prone position (*P* ≤ 0.008 for both). C_RS_ improved by a mean of 5.2 ± 3.4 mL/cmH_2_O in the supine position and 3.6 ± 3.2 mL/cmH_2_O in the prone position (*P* ≤ 0.004 for both).

### Increasing V_T_ and PEEP

In 6 patients, increasing V_T_ and/or PEEP resulted in an immediate rise of peak airway pressures to above 50 cmH_2_O; in these cases, attempts to measure airway pressure after 5 tidal breaths had been delivered were not pursued out of concern for patient safety.

V_T_ was increased in twelve patients. An increased DP was observed in eleven of these patients; reduced C_RS_ was observed in eight. In response to the V_T_ increase, DP increased by a mean of 5.5 ± 5.7 cmH_2_O in the supine position and 3.0 ± 1.3 cmH_2_O in the prone position (*P* = 0.03 supine; *P* < 0.001 prone). C_RS_ deteriorated by a mean of 1.2 ± 4.1 mL/cmH_2_O in the supine position and 1.1 ± 2.8 mL/cmH_2_O in the prone position (*P* = 0.44 supine; *P* = 0.32 prone) (Additional file 1 Table S1).

Holding V_T_ unchanged, PEEP was increased in fourteen patients; increased DP and reduced C_RS_ were observed in ten of these. In the remaining four, two had no change in DP and C_RS_, and two had subtle improvements. The PEEP increment caused DP to increase by a mean of 3.4 ± 4.6 cmH_2_O in the supine position and by 1.0 ± 1.2 cmH_2_O in the prone position (*P* = 0.04 supine; *P* = 0.07 for prone). C_RS_ deteriorated by a mean of 2.2 ± 2.2 mL/cmH_2_O in the supine position and 1.7 ± 2.4 mL/cmH_2_O in the prone position (*P* = 0.01 supine; *P* = 0.11 prone) (Supplemental Table 1).

## Discussion

Among our cohort of patients with moderate-severe ARDS who demonstrated a paradoxical response to manual chest wall loading (17/19), small changes in tidal volume or PEEP exerted strong effects on C_RS_ and DP, despite the intent by their caregivers to follow ‘lung protective’ guidelines to the extent consistent with adequate gas exchange. The presence of mechanical paradox was associated with reduced C_RS_ at baseline; almost all (15/17) paradox positive patients had a baseline C_RS_ < 40 mL/cmH_2_O, and the majority (11/17) had a baseline C_RS_ ≤ 30 mL/cmH_2_O. Assuming end-tidal overdistension as the most likely explanation for ‘paradox’, this finding is not unexpected. Because the low compliance state of ARDS reflects primarily the reduced capacity of the ‘baby lung’, as opposed to altered elastic properties of remaining functional lung units [9], the risk of end-tidal overdistension would be expected to rise as compliance declines [10]. The severity of lung disease in our patient sample is also reflected by the CO_2_ elimination data of Table 2 (PaCO_2_ and ventilatory ratio).

Interestingly, the presence of paradox itself did not correlate well with duration of mechanical ventilation or hospitalization. While mean duration of hospitalization and of intubation were 16.8 and 11.2 days, respectively, at the time of data collection, over half (9/17) of our patients had been intubated for fewer than six days, and almost one third (5/17) had data collected within one day of intubation.

Consistent with our hypothesis that interventions alleviating end-tidal overdistension would lead to improved mechanics, there was universal improvement in C_RS_ following a minor decrease in either V_T_ or PEEP from the baseline value. Conversely, there was a clear trend toward increased DP and reduced C_RS_ following increases of either machine setting. Indeed, extreme rises in airway pressures in response to small increments of V_T_ or PEEP prevented data from being collected in several patients out of concern for safety; as a result, our data understate the adverse response in our patients with mechanical paradox to increasing either V_T_ or PEEP.

All patients in this study were receiving ventilation with low V_T_ and low to moderate levels of PEEP at baseline; mean V_T_ was 5.6 ± 0.9 mL/kg PBW and mean PEEP was 9.7 ± 3.2 cmH_2_O. Nonetheless, decreasing V_T_ by 1 mL/kg PBW or PEEP by 2.5 cmH_2_O resulted in a disproportionate reduction in DP and, therefore, improved C_RS_. These findings are consistent with those of prior studies demonstrating radiographic evidence of significant hyperinflation in as many as one third of patients treated with a ventilatory strategy targeting 6 mL/kg PBW V_T_ and P_plat_ < 30 cm H_2_O [11]; they further demonstrate that no generalized ventilatory strategy, even those generally considered ‘lung-protective,’ can be employed indiscriminately without further concern regarding safety for the individual under care.

The paradoxical response to chest wall loading, in which C_RS_ unexpectedly improves following a decrease in chest wall volume, has been described in several recent reports. In these cases, C_RS_ improved not only in response to direct compression of the chest wall [3, 4, 6], but also in response to interventions that resulted in cephalad displacement of the diaphragm, including abdominal compression [2, 5]; compression of the lumbar region (while prone) [1]; and placement in a less upright position [2, 4, 5]. Considered collectively and in association with the data reported here regarding ventilatory pattern, the most plausible unifying explanation for mechanical paradox is that tidal ventilation infringes on the upper flat portion of the lung’s pressure volume inflation curve. Compression of the chest wall results in a forced volume reduction of lung units otherwise overdistended at end inspiration, leading to descent along the pressure–volume curve to a position more favorable to tidal excursions [12]. Limited data from studies that have used electrical impedance tomography and computed tomography with quantitative density analysis support this hypothesis, even though the precise mechanism remains unconfirmed [4, 6]. An alternative explanation may be that, in the setting of ARDS, heterogeneity gives rise to *unaltered* lung units that are buttressed by zones of inflammatory debris and edema; as a result, these fortified lung units may be exposed to high transpulmonary pressures without being subjected to injurious strain. In this scenario, volume reduction of such units may still lead to improved C_RS_, but without the same implications regarding end-tidal overdistension of those embedded individual units.

### Limitations

While these findings suggest end-tidal overdistension in patients with mechanical paradox, our study was not designed to be mechanism defining, but rather to focus selectively on the diagnostic value of detecting a paradoxical response to chest wall compression as it pertains to the ventilatory prescription. As such, we did not measure esophageal pressure for the purpose of partitioning C_RS_ into its individual components (C_CW_ and C_L_). For safety concerns, changes in V_T_ or PEEP were sustained only for brief periods of time and, as noted, in several cases not pursued when small increases were attempted. Although we observed no decreases in oxygen saturation during any loading maneuver or parameter change, the effects of altering the ventilatory prescription on gas exchange or hemodynamics could not be evaluated.

We did not perform interventions in a randomized sequence, primarily because we felt that doing so would compromise the consistency and efficiency of data collection; in each case, however, the duration of alterations was short lived, and the baseline was restored between interventions. As such, we think it is unlikely that randomizing V_T_ and PEEP would have had a significant effect on our findings. Our sample was also drawn primarily from patients affected by C-ARDS, many of whom had been intubated for over a week. Therefore, our results might differ quantitatively (but we suspect not qualitatively) from patients with other forms of severe ARDS. Finally, we emphasize that no conclusions can be drawn regarding the effect of decreasing V_T_ or PEEP on clinical outcomes of paradox positive patients on the basis of our findings.

### Clinical implications

Repeated exposure to tidal cycles that cause excessive strain of lung parenchyma is believed to be a proximate stimulus for ventilator-induced lung injury in ARDS [13]. Once a strain threshold is exceeded and mechanical forces disrupt structural microelements, previously functioning lung units will begin to drop out, initiating a positive feedback cycle whereby inflation energy (and power) concentrate among fewer and fewer units [10]. Lung heterogeneity exacerbates this process further, leading to the amplification of stress at the interface of open and closed lung units [14]. Tidal volumes operating in the ‘upper inflection zone’ not only encourage damaging strain—both global and regional, but also risk barotrauma, regional small airway remodeling, and distortion of vulnerable lung units that are hyperinflated at end-inspiration. Such mechanical processes may help explain the highly regionalized emphysematous changes [15], noteworthy incidence of pneumothorax [16], and rapidly evolving bronchiectasis encountered in C-ARDS [17].

In our study, not only was mechanical paradox encountered in all but two of the nineteen patients with ARDS who met our inclusion criteria, but also it was present in spite of consistent adherence to ventilator settings widely regarded as lung-protective; it was frequently encountered early in the course of invasive mechanical ventilation; and the mechanics of all patients with paradox responded favorably to even small reductions in V_T_ and PEEP. The use of manual compression to detect paradox may thus serve as a valuable tool for revealing otherwise undetected excessive tidal strain, and its presence should prompt re-evaluation of modifiable determinants of end-tidal overdistension, including PEEP, V_T_, and positioning [18, 19]. In some patients with severe and unresolving ARDS, however, protection of the entire lung may simply be impossible without extracorporeal gas exchange as excessive end-tidal strain may be the unavoidable consequence of adequate ventilation.

## Conclusions

A paradoxical response to chest wall loading is frequently observed in the setting of moderate to severe ARDS, particularly in the setting of low C_RS_. Our data demonstrate that paradox can be present early in the course of mechanical ventilation and occur despite conservative application of V_T_ and PEEP. Paradox-positive patients demonstrate uniform improvement of C_RS_ following minor reduction in either V_T_ or PEEP, findings in keeping with prior evidence suggesting that paradox is a sign of tidal overdistension.

## Supplementary Information

**Additional file 1: Table S1 **Response to increased tidal volume and positive end-expiratory pressure V_T_ was increased by 1 mL/kg PBW in a total of twelve patients (baseline values for these twelve patients in column A); PEEP was increased by 2.5 cmH_2_O in a total of fourteen patients (baseline values for these fourteen patients in column B). *DP* driving pressure, *C*_*RS*_ system compliance, *V*_*T*_ tidal volume, *PEEP* positive end-expiratory pressure.

## Data Availability

The datasets used and/or analyzed during the current study are available from the corresponding author on reasonable request.

## Abbreviations

ARDS: Acute respiratory distress syndrome
C-ARDS: Acute respiratory distress syndrome secondary to COVID-19
C_L_: Lung compliance
C_RS_: Respiratory system compliance
C_CW_: Chest wall compliance
DP: Driving pressure
PEEP: Positive end-expiratory pressure
PBW: Predicted body weight
V_T_: Tidal volume
