# Supplementary material for: Paradoxical response to chest wall loading predicts a favorable mechanical response to reduction in tidal volume or PEEP
Source: Crit Care. 2022 Jul 5;26:201. doi: 10.1186/s13054-022-04073-2 (PMC9255488; doi:10.1186/s13054-022-04073-2)
Supplement: Supplementary file 1 — Additional file 1: Table S1 Response to increased tidal volume and positive end-expiratory pressure VT was increased by 1 mL/kg PBW in a total of twelve patients (baseline values for these twelve patients in column A); PEEP was increased by 2.5 cmH2O in a total of fourteen patients (baseline values for these fourteen patients in column B). DP driving pressure, CRS system compliance, VT tidal volume, PEEP positive end-expiratory pressure. [file 13054_2022_4073_MOESM1_ESM.docx]

|  |  | Baseline 1  (A) | Baseline 2  (B) | ↑ V_T_  (C) | ↑ PEEP  (D) | A to C  (P value) | B to D  (P value) |
| --- | --- | --- | --- | --- | --- | --- | --- |
| Supine | DP  (cmH_2_O) | 14.6 ± 6.2 | 16.1 ± 7.3 | 20.1 ± 11.8 | 19.5 ± 9.9 | 0.03 | 0.04 |
|  | C_RS_  (mL/cmH_2_O) | 30.5 ± 12.8 | 28.3 ± 12.3 | 29.3 ± 15.6 | 26.1 ± 13.3 | 0.43 | 0.01 |
| Prone | DP  (cmH_2_O) | 13.4 ± 3.4 | 13.4 ± 3.4 | 16.4 ± 3.9 | 14.3 ± 3.8 | 0.0009 | 0.07 |
|  | C_RS_  (mL/cmH_2_O) | 29.2 ± 10 | 29.2 ± 10 | 28 ± 9.4 | 27.5 ± 9.8 | 0.32 | 0.11 |

**Supplemental Table 1 Response to increased tidal volume and positive end-expiratory pressure** V_T_ was increased by 1 mL/kg PBW in a total of twelve patients (baseline values for these twelve patients in column A); PEEP was increased by 2.5 cmH_2_O in a total of fourteen patients (baseline values for these fourteen patients in column B). *DP* driving pressure, *C_RS_* system compliance, *V_T_* tidal volume, *PEEP* positive end-expiratory pressure.
